# The impact of economic policy uncertainty on firms’ investment in innovation: Evidence from Chinese listed firms

**DOI:** 10.1371/journal.pone.0272983

**Published:** 2022-11-15

**Authors:** Wen Kun, Xiangxiang Hu, Fajiang Liu

**Affiliations:** 1 Hainan Vocational University of Science and Technology, Haikou, China; 2 Jiangxi Administration Institute, Nanchang, China; 3 School of Economics and Management, Hunan University, Changsha, China; Universidad Nacional Autonoma de Nicaragua Leon, NICARAGUA

## Abstract

This paper uses data of Chinese listed enterprises and economic policy uncertainty index for empirical analysis, and conducts a study through three channels of monetary policy uncertainty affecting enterprise innovation investment, and finds that economic policy uncertainty has a positive promotion effect on enterprise R&D investment, and its increase in tension is instead a clear signal that can effectively increase enterprise R&D investment, this promotion effect seems unexpected, this paper Through theoretical analysis and combined with the actual practice, this incentive effect is found to be in line with reality. However, in the subsequent heterogeneity analysis, this paper finds that it positively promotes R&D investment when economic policy uncertainty is low and may have a suppressive effect on R&D investment when monetary policy uncertainty is high.

## 1. Introduction

Since the reform and opening-up, China’s GDP growth rate has been on the rise. Still, in recent years China has entered a bottleneck in its development. Its economic growth rate is at a relatively low point [1]. China has entered a critical transition period, where innovation has become the focus of financial work and the first driving force of economic development [2–4]. Enterprises are important micro-entities for implementing innovation strategies. Enterprise-led innovation enhances profits and enterprise value and is the primary implementation vehicle for China’s high-quality economic development strategy. Currently, governments at all levels are actively implementing reform measures through decentralization and deepening reforms to promote enterprises to tap their innovation potential and increase their innovation momentum to facilitate the transformation of the whole society to high-quality development [5, 6]. However, the government’s proactive actions have increased economic policy uncertainty while giving signals to the market to move forward [7]. Therefore, in the current situation where the economic growth rate is relatively low, China faces a high monetary policy uncertainty. Innovation is placed at the core of economic development, so the impact of economic policy uncertainty on firms’ innovation investment and R&D investment is crucial [8, 9]. The following paper will theoretically analyze and empirically test the impact of economic policy uncertainty on firms’ innovation investment and its channels.

This paper starts from the channel of influence and argues that the degree and direction of the impact of economic policy uncertainty on corporate innovation through different channels are different, and there is an interactive effect. Therefore, this paper does not directly hypothesize the impact of EPU on innovation, but instead, it looks at the three main factors that affect investment—investment return, waiting for value, and investment costs, to explore the impact of economic policy uncertainty on innovation through various channels. The main contributions of this paper are to provide empirical evidence on the impact of economic policy uncertainty on firms’ innovation investment in China and provide relevant suggestions for policy-making authorities. The government has been using monetary policy as the primary intervention tool in China’s ongoing exploration of its economic system; in addition, China has experienced high economic growth and relatively high returns to business investment over the past years [10], so the impact of monetary policy uncertainty on firms’ innovation inputs may differ significantly between China and developed economies. Second, this paper starts from the channel of influence and argues that the degree and direction of the impact of economic policy uncertainty on firms’ innovation investment through different channels are different. There is an interaction effect, so this paper does not directly analyze and hypothesize the relationship between the two variables. Still, instead, it looks at the three main factors that affect investment—Therefore, this paper does not directly analyze and hypothesize the relationship between the two variables but explores the impact of economic policy uncertainty on innovation input through various channels, starting from the three main factors affecting investment—investment return, waiting for value, and investment cost, and finally analyzing the overall impact of economic policy uncertainty on innovation input. Third, although the full-sample regression results of this paper show that monetary policy uncertainty positively promotes R&D investment of firms, this paper finds in the subsequent heterogeneity analysis that it simply encourages R&D investment when economic policy uncertainty is low and may have a suppressive effect on R&D investment when monetary policy uncertainty is high, which enriches the empirical findings in this area and provides future related literature to test the non-linear relationship between the two and offer research directions for future related literature.

## 2. Literature review

### 2.1. Economic policy uncertainty

There is still no consensus in the relevant literature on how economic policy uncertainty should be measured. Existing studies use three main types of methods: (1) dummy variables for the occurrence of political events such as policies or general elections [11]; (2) data on official changes at the national or local level [12]; and (3) the EPU index, constructed by [13] as an economic policy uncertainty index. Among the above three indicators, political events and official turnover data are relatively strictly exogenous from other economic variables. Still, they suffer from disadvantages such as discontinuity in the sample period and time lag [14]. The EPU index constructed by [13] can effectively overcome these two shortcomings and reflect economic policy uncertainty more accurately. The EPU index is also used in the subsequent empirical analysis of this paper, which more accurately and scientifically quantifies the overall uncertainty of the monetary, fiscal, and tax policies officially introduced by the government.

### 2.2. The impact of economic policy uncertainty on firms’ investment in innovation

The negative impact of various types of economic policy uncertainty on innovation investment has been studied in several pieces of literature. [15] argues through a normative analysis that the technological lag in innovation in some traditional industries is caused by economic policy uncertainty. [16] analyzed the impact of trade policy uncertainty on Chinese export-oriented firms by cutting from trade policy and found that its fate is significantly and negatively related to firms’ product innovation. [17] found that changes in official policies make firms unsure how to adapt, add unnecessary impediments, raise the cost of innovation, lead to weaker incentives to innovate, and adversely affect R&D expenditures, which is particularly pronounced for innovation-intensive firms [18]. On the other hand, it uses data on municipal secretary changes to quantify policy uncertainty. The empirical results find that uncertainty from municipal secretary turnover negatively affects firms’ patent numbers and innovation efficiency through the financing constraints and cash flow uncertainty channels. [11] using Chinese data, found that uncertainty, whether on the policy side or the market side, negatively affects firms’ innovation investment.

Although real options theory emphasizes that high switching costs and irreversibility can lead firms to postpone R&D investments in the face of heightened uncertainty, it is typically based on two specific assumptions [19]: that (1) firms have a monopoly on investment opportunities and (2) their behavior does not affect prices or market structure. The actual market does not conform to either of these, so the impact of uncertainty on firms’ innovation investments will vary. Subsequent theoretical research explores several other influences and mechanisms that may limit firms’ waiting and promote innovative investment.

The first is that risks and opportunities coexist and are often positively correlated with returns. Economic policy uncertainty means that while there are risks in investment projects, there are also implied opportunities to seize the market, and the value of entrepreneurship lies in the ability to scientifically judge and correctly understand, through the investment risks in an uncertain environment, the potential investment opportunities to realize economic returns and corporate value. Therefore, the rise in monetary policy uncertainty implies the opportunity for increased future returns, which can effectively lead to increased motivation to invest in R&D [20]. The second is that competitors influence the value of waiting. Since R&D investments cannot be made independently of corporate strategy [21], when firms choose to stay, competitors may complete similar products first and thus may lose the opportunity to capture the market. As a result, the waiting value of R&D projects will be reduced or zero [22], and the project’s overall value will be seriously eroded [23]. The third is the growth option, which provides firms with future "upside potential" [24]. While it is true that economic policy uncertainty can hurt innovation investment due to the irreversibility of investment, from another perspective, the high technological uncertainty and long investment time of R&D projects create valuable call options for investment, i.e., after the initial investment, more investment opportunities in the future relative to other competitors, which allows firms to gain competitive advantages that are much larger than the negative ones. This will enable firms to gain competitive advantages that are much greater than the negative ones, which drives forward R&D investments [25].

### 2.3. Literature review

There are two shortcomings in the current literature: (1) most of the existing studies adopt the real options theory of analyzing the influence mechanism between the two and consider R&D investment as one of the essential components of corporate investment. Although other scholars have improved the view from market competition and growth options, it is still based on the fundamental options theory. And the author believes that innovation investment has the characteristics of sticky investment and lagging returns. It is impossible to measure the sum of current returns, investment costs, and waiting value, so innovation investment does not apply to the fundamental options theory. (2) The existing literature mostly stays on the correlation between the two, and the empirical research on the influence mechanism is not sufficient. Even if there is, it only judges the magnitude of the regression coefficient of EPU index on innovation investment in two types of sub-samples (such as high or low financing constraints, whether it is a state-owned enterprise, whether it is a manufacturing industry, etc.). However, the author believes that, firstly, this empirical method does not introduce interaction terms, and the sub-sample regressions do not well elucidate the impact channels of economic policy uncertainty; secondly, theoretically speaking, these groupings are based on the nature of enterprise property rights, the industries they are in, etc., which are non-continuous variables that the external macro policy environment cannot change, and at most, we can only assume that the impact generated by economic policy uncertainty exists between the two types of enterprises At most, it can be argued that the effects of monetary policy uncertainty are heterogeneous between two kinds of firms. These firm characteristics cannot be considered as channels of influence.

To address the shortcomings of the above literature, the critical innovation of this paper is that the degree and direction of the impact of economic policy uncertainty on firms’ innovation investment through different channels are different. Therefore, this paper does not directly hypothesize the law of the relationship between the two but starts from the main factors affecting investment—investment return, waiting for value, and investment cost—to explore the impact of economic policy uncertainty on innovation investment through various channels, which has both positive and negative effects, and finally analyzes the overall development of monetary policy uncertainty on innovation investment. Finally, we analyze the overall impact of policy uncertainty on innovation investment.

## 3. Theoretical analysis and research hypothesis

### 3.1. EPU affects the channel of investment returns on firms’ innovation inputs—Cash flow uncertainty

According to fundamental options theory, for public investments, cash flow uncertainty represents a better investment opportunity in the future, thus increasing the waiting value of the investment, and therefore firms will delay the acquisition. However, the fundamental option theory is not very applicable to innovation investments because of the lagged return feature, which does not produce immediate returns at present. For innovation investment, cash flow uncertainty is the only source of profit [26], which is positively correlated with future returns and represents to some extent the potential value and growth space of the firm and can motivate firms to favor R&D investment in project decisions and increase the intensity of financial support for innovation projects [20]. Companies with foresight, economic intuition, and strong business capabilities are more likely to see through the apparent risks and seize potential opportunities and have the ability and willingness to bear the possible negative impacts of R&D investments, so cash flow uncertainty has an incentive effect on innovation investment by such companies. In addition, listed companies are generally well-capitalized, robust, with high market share, and forward-looking, so the "winner effect" is usually more substantial. Thus the impact of cash flow uncertainty on innovation investment is mainly reflected in the "funding effect."

Therefore, this paper argues that when EPU rises, cash flow uncertainty increases, and firms increase innovation to enhance their long-term earnings, thus increasing R&D investment. This leads to the following hypothesis. **Hypothesis 1: An increase in economic policy uncertainty raises firms’ cash flow uncertainty, thus boosting their R&D investment.**

### 3.2. EPU influences the waiting value channel of innovation input of the company—Product market competition

Classical real options theory suggests that an increase in EPU will increase an investment project’s "wait" value. However, we need to note that in the real world, the waiting value of a firm’s innovation activities will also be influenced by the behavior of its competitors. A key feature of R&D investments is that they cannot be made independently of a firm’s strategy [19]. When a firm chooses to wait, competitors may complete the development of similar products first. Thus the firm may lose the opportunity to capture the market, and the value of waiting will be reduced, or zero [22], and the importance of R&D projects will be severely eroded. If a company is in the midst of a transition or upswing, and the current investment opportunity is critical to the overall growth. Competitors are "eyeing" the project, then "prudent" investment is no longer the optimal solution at the moment. When the economic policy uncertainty is high, each competitor is in a state of indecision when faced with the fate of revenue. At this time, companies with subjective solid motivation and awareness of opportunities consider it an excellent time to seize the opportunity and seize the first opportunity to increase market competitiveness [27], so the economic policy uncertainty intensifies the degree of competition in the product market.

As one of the main external environments of enterprises, product market competition has a positive, stimulating effect on innovation. First of all, product market competition can stimulate the internal innovation motivation of enterprises. In a competitive environment with serious homogeneity and countless rivals, enterprises have to increase innovation if they do not want to be buried by the tide of the times. On the one hand, they can achieve cost reduction through intelligent technology. On the other hand, they can break the homogeneity of the market through new products, establish competitive advantages with differentiated products, broaden business channels and market demand, and turn passive defense into the active competition. Secondly, product market competition can force enterprises to improve their innovation ability. Because of the solid positive externality of manufacturing product technology, market competition will bring a knowledge spillover effect, favorable for relatively backward enterprises, i.e., enterprises can use it to acquire knowledge of technology. In addition, innovation projects will require the input of R&D talents. This talent demand drives the ability to invest in talent resources and the construction of related infrastructure in the industry.

Thus, economic policy uncertainty increases competition in the market, reduces the value of "waiting," and severely erodes the value of R&D projects, thus encouraging firms to invest in innovation earlier. This leads to the following hypothesis. **Hypothesis 2: An increase in economic policy uncertainty increases competition in the product market and thus promotes R&D investment.**

### 3.3. EPU affects the investment cost channel of innovation input of enterprises—Debt financing

The increase in economic policy uncertainty will reduce corporate debt financing. Firstly, from the perspective of borrowing firms, the growth in monetary policy uncertainty makes it difficult for firms to make scientific and reasonable forecasts of future economic trends, forcing them to hold back on project investments and observe the situation. Therefore their debt financing needs arising from investment projects are massively curtailed. In addition, enterprises also increase their cash holdings and reduce their marginal values [28], leading to higher uncertainty in future operations, making the chances of not being able to repay on time in a coming period much higher even if enterprises apply for loans. Furthermore, to avoid the default above risk and bankruptcy risk, enterprises usually also actively reduce the scale of debt financing, so the overall debt financing of enterprises will weaken enterprises’ available debt financing opportunities. Secondly, from the bank’s perspective, economic policy uncertainty, as a systemic risk that is difficult to avoid, directly affects the banking system, leading to a rise in non-performing bank debt. Therefore, for self-protection purposes and profitable business philosophy, banks will scale back credit. In addition, external uncertainty also makes banks’ credit assessment and lending decisions much more difficult [29], so they may impose higher interest rate costs and more demanding and complicated conditions, such as lower credit limits, longer approval times, and stricter approval conditions [30], causing firms to suffer from severe financing constraints [31]. In summary, it is not difficult to conclude from both the supply and demand sides that corporate debt financing decreases as economic policy uncertainty increases.

As for the impact of the level of debt financing on firms’ R&D investment, on the one hand, the reduction of debt financing directly leads to the decrease in firms’ innovation investment; on the other hand, firms will construct optimal solutions in terms of investment decisions according to the macro environment they are in and their internal operations. Therefore, in the face of different degrees of economic uncertainty, even if they receive the same total amount of financing, the allocation on various investment activities is quite different. When policy uncertainty increases, firms’ financed funds are more likely to be used to replenish liquidity, for routine expenditures, and maintain financial soundness [32], leading to a less positive effect of financing on investment. This leads to the following hypothesis. **Hypothesis 3: Increased economic policy uncertainty reduces the level of corporate debt financing and thus inhibits corporate R&D investment.**

## 4. Study design

### 4.1. Data source

The economic policy uncertainty index is obtained from the Economic Policy Uncertainty website, and other financial data are obtained from the CSMAR database. The initial sample of this paper is selected from all A-share listed enterprises in China from 2012 to 2020. For statistical research, the initial sample data are processed as follows: (1) excluding financial enterprises specified by the industry 2012 classification standard of the Securities and Futures Commission; (2) excluding ST and ST* enterprises based on the information of previous years; (3) excluding enterprises with missing data, discontinuous sample years, etc.; (4) applying 1% tailoring to the sample of continuous variables at the enterprise financial level. (3) eliminating enterprises’ data with missing data and discontinuous sample years; (4) applying a 1% tailing process to the sample of continuous variables at the enterprise financial level. The paper is finally screened to 2378 firms, and an unbalanced panel of 13435 firm-annual observations is obtained.

### 4.2. Variables

The explanatory variables are firm innovation inputs, and the common indicators used to measure vital innovation in the existing literature are R&D inputs and patent applications. However, patent application reflects the resultant output of innovation activities. Therefore, it lags behind the initial R&D input behavior of corporate innovation projects, i.e., patents applied for in the current period are the result of previous R&D inputs and do not reflect the immediate reflection of recent economic policy uncertainty. In contrast, R&D input demonstrates the change of corporate investment strategy in response to monetary policy uncertainty. Therefore, this paper adopts R&D investment (R&D) as the explanatory variable.

The core explanatory variable in this paper is economic policy uncertainty (EPU), and the monetary policy uncertainty index constructed by [13] is used. The index is built using the South China Morning Post as the data construction source and indexing platform. The newspaper’s frequency of articles about economic policy uncertainty is calculated based on statistical methods such as information filtering and text indexing EPU index in China. The EPU index accurately and scientifically quantifies the overall tension of the government’s official economic policies such as monetary, fiscal, and taxation policies. In this paper, the month *EPU* is transformed into an annual *EEU* by taking a weighted average of the monthly *EPU*_*tj*_ by Eq (1) [22] *PU*, the larger the value of this variable, the more unstable the economic policy faced. In the subsequent robustness test of this paper, the arithmetic mean is also taken to obtain the annual *EPU* for regression.


$$EPU_{t}=\frac{1}{78}*EPU_{t1}+\frac{2}{78}*EPU_{t2}+\frac{3}{78}*EPU_{t3}+\cdots +\frac{12}{78}*EPU_{t12}$$ (1)

### 4.3. Influence channel variables

The first impact channel variable in this paper is cash flow uncertainty (CFV), which is broadly measured by existing literature using three types of indicators: (1) financial-type indicators, (2) volatility indicators, and (3) value-at-risk indicators. In this paper, we use the volatility indicator of the standard deviation of ROA for the last three years to measure cash flow uncertainty.

The second influence channel variable in this paper is product market competition (HHI), which adopts the Herfenind index commonly used in the existing literature to reflect the degree of product market competition as well as market concentration as: *HHI*_*j*_ = Σ(*x*_*i*_/*x*_*j*_)^2^, where *x*_*j*_ is the total operating revenue of industry j. It is the operating revenue of firm i in industry j. The HHI index effectively reflects the degree of market concentration and product market competition in the industry. The value of the index is positively proportional to the degree of market concentration and inversely proportional to the degree of market competition.

The third channel of influence variable in this paper is debt financing (Debt), broadly measured in two ways in the existing literature. One is the increase in total liabilities, and the other is the increase in interest-bearing debt. The former covers the obvious debt contract financing methods and a variety of implicit financing such as accounts payable, bonds payable, notes payable, etc. Therefore, this paper uses "increase in total liabilities/total assets at the beginning of the period" to measure debt financing.

### 4.4. Control variables

The control variables selected in this paper are shown in Table 1. they are cash flow (CF), firm age (Age), firm size (Size), sales size (Sale), gearing (Lev), return on assets (ROA), proportion of tangible assets (Tangibility), TobinQ, and GDP growth rate (DGDP).

**Table 1:** Definition of important variables.

Variable Type	Variable Name	Variable Symbols	Variable Meaning
Explained variables	R&D input	*R*&*D*	R&D investment/business revenue
Significant explanatory variables	Economic policy uncertainty	*EPU*	Annual weighted arithmetic average of China EPU Index, divided by 100
	Cash flow uncertainty	*CFV*	The standard deviation of ROA for the last three fiscal years
	Product market competition	*HHI*	The sum of the squares of the percentages of the industry’s total revenue accounted for by the leading business revenue of each business entity in the industry
	Debt financing	*Debt*	(Total liabilities at the end of the period—Total liabilities at the beginning of the period)/Total assets at the beginning of the period
Control variables	Cash flow	*CF*	Operating cash flow/total assets at the beginning of the period
	Company age	*Age*	Natural logarithm of the number of years the company has been in operation since its establishment
	Company size	*Size*	Natural logarithm of the company’s total assets
	Sales size	*Sale*	Natural logarithm of operating income
	Gearing Ratio	*Lev*	Company’s total liabilities/total assets
	Return on Assets	*ROA*	Net profit / average balance of assets
	Tangible Assets Ratio	*Tang*	Tangible assets/total assets
	Tobin’s Q value	*TobinQ*	(Company’s liquid market value + company’s non-liquid market value + book value of liabilities)/book value of assets
	GDP growth rate	*DGDP*	(Current GDP—Previous GDP) / Previous GDP

### 4.5. model construction

To test the impact of economic policy uncertainty on R&D investment through the cash flow channel, referencing [33], model (2) is constructed.


$$R\&D_{it}=\beta_{0}+\beta_{1}*EPU_{t-1}+\beta_{2}*CFV_{it-1}+\beta_{3}*EPU_{t-1}*CFV_{it-1}+\Sigma \gamma Control_{it-1}+\alpha_{j}+\mu_{t}+\varepsilon_{it}$$ (2)

The explanatory variable is R&D investment (R&D), the core explanatory variable is the interaction term between economic policy uncertainty and cash flow uncertainty ("*EPU*_*t*−1_*CFV_*it*−1_"), and the control variables and their definitions are shown in Table 1. α is an industry effect and μ is an annual effect. All explanatory variables in this paper are lagged by one period. The interaction term is used to test the mechanism of the impact of economic policy uncertainty by increasing the tension of firms’ cash flows and thus promoting R&D investment. According to Hypothesis 1, it is expected that *β*_3_>0.

To test the effect of economic policy uncertainty on R&D investment through the product market competition channel, model (3) is constructed in this paper.


$$R\&D_{it}=\beta_{0}+\beta_{1}*EPU_{t-1}+\beta_{2}*HHI_{jt-1}+\beta_{3}*EPU_{t-1}*HHI_{jt-1}+\Sigma \gamma_{i}Control_{it-1}+\alpha_{j}+\mu_{t}+\varepsilon_{it}$$ (3)

The dependent variable is R&D investment. The core explanatory variable is the interaction term between economic policy uncertainty and product market competition ("*EPU*_*t*−1_*HHI_*it*−1_"), which is used to explore the mechanism by which monetary policy uncertainty induces firms to invest in R&D earlier by increasing product market competition. Therefore, according to Hypothesis 2, *β*_3_<0 it is expected.

To test the effect of economic policy uncertainty on R&D investment through the debt financing channel, model (4) is constructed.


$$R\&D_{it}=\beta_{0}+\beta_{1}*EPU_{t-1}+\beta_{2}*Debt_{it-1}+\beta_{3}*EPU_{t-1}*Debt_{it-1}+\Sigma \gamma Control_{it-1}+\alpha_{j}+\mu_{t}+\varepsilon_{it}$$ (4)

The dependent variable is R&D investment (R&D), and the core explanatory variable is the interaction term between economic policy uncertainty and debt financing ("*EPU*_*t*−1_*Debt_*it*−1_"), which is used to test the mechanism of the effect of monetary policy uncertainty by curtailing the size of debt financing and thus inhibiting firms’ R&D investment. Therefore, according to Hypothesis 3, *β*_3_<0 it is expected.

## 5. Results and discussion

### 5.1. Baseline regression

We used STSTA software (version: Stats for Mac 2.6.22) for data collation and modeling analysis. First, this paper does not include the three influential channel variables. Instead, it runs the regression with corporate R&D investment as the explanatory variable and the EPU index as the primary explanatory variable. Model (3) in Table 2 demonstrates that the coefficient of the EPU variable is 0.0017 indicating that there is a significant positive relationship between economic policy uncertainty and corporate R&D investment, so overall, an increase in monetary policy uncertainty is instead a positive signal for corporate R&D, effectively promoting corporate R&D investment. In terms of economic significance, for every one standard deviation increase in the China EPU index, the average increase in enterprise R&D investment is 0.49.

**Table 2:** Impact of economic policy uncertainty on firms’ investment in innovation.

Dependent variable: *R*&*D*	(1)	(2)	(3)
*EPU*	0.0016***	0.0024***	0.0017***
	-4.669	-3.4696	-4.1297
*CF*		0.0011**	0.0007**
		-2.6496	-2.4197
*Size*		0.0136***	0.0154***
		-3.1296	-3.8797
*Age*		-0.0135***	-0.0083***
		-4.3396	-4.2497
*Sale*		-0.0164***	-0.0156***
		-4.5896	-4.7897
*Lev*		-0.0369***	-0.0296***
		(-3.82)	(-4.94)
*Tang*		-0.0189	-0.0082
		(-1.40)	(-1.17)
*TobinQ*		0.0050**	0.0037***
		-2.6596	-3.1997
*ROA*		-0.006	0.0066
		(-0.49)	-0.8297
*DGDP*		-0.1162	-0.1479**
		(-0.99)	(-2.45)
Constants	0.0383***	0.1559***	0.0559**
	-6.769	-5.1096	-2.4297
Industry effect	No	No	Yes
Year effect	No	No	Yes
Sample size	13435.001	13435.0004	13435.0003
R^2^	0.004	0.2164	0.4373

Note: Values in parentheses are t-values

***, **, * indicate significant at 1%, 5%, 10% confidence level, respectively.

From the results in the above table, it can be seen that given other variables unchanged, economic policy uncertainty will have a promoting effect on the innovation investment of enterprises, which verifies the point of view of the promoting effect in the mechanism path analysis, indicating that economic policy uncertainty Although the debt problem and market signal screening problem will inhibit the innovation investment of enterprises, considering the investment substitution in the context of economic policy uncertainty, increasing innovation investment will easily bring first-mover advantages and competitive advantages to enterprises, and R&D There is dynamic continuity in the level of investment. Hence, enterprises are more inclined to increase investment in innovation capital and personnel, indicating that economic policy uncertainty will help improve the level of innovation investment. This result is contrary to the conclusions of some literature studies, which believe that the corporate R&D process has the characteristics of a long time and unpredictable results, as well as a high risk of failure so that policy uncertainty will inhibit R&D investment [34, 35]. There are several reasons why this paper has different results from the above conclusions. First, under the conditions of the uncertain market environment, the initial R&D investment of the enterprise can obtain future growth options and give new opportunities for the future development of the enterprise, which can prevent new competitors from entering or prompt competitors to make concessions, thereby obtaining Competitive advantage in the market [27]. Second, when economic policy uncertainty rises, companies may reduce their initial physical investment plans and invest related funds in the field of corporate innovation [3], and higher uncertainty will prompt companies to invest in innovation, that is, economic policy uncertainty can instead promote innovative activities of firms [6]. Finally, since enterprise innovation investment is a continuous and dynamic process, excessive economic policy uncertainty will lead to extreme external operating environment uncertainty. Enterprises abandoning existing R&D investments will lead to high sunk costs.

In addition to the above reasons, in the unique environment of the Chinese market, the positive effect of EPU on R&D investment seems to be somewhat unexpected. However, through the above analysis, and based on China’s current economic situation, it is not difficult to analyze them. Reason: In the past, there was a big gap between China and the world’s cutting-edge technology. Enterprises only needed to imitate technology through the spillover effect of knowledge. Then they could enter the market at a lower cost, which caused severe homogeneity of enterprises and intensified product market competition [28]. At present, accelerating the innovation of products and technologies has become the only rule for companies not to be eliminated or even to stand out in the industry that competes with others. Against such a competitive background, economic policy uncertainty brings challenges and means opportunities to increase earnings in the future. Although it negatively affects the general and physical capital investment of enterprises, it is rational and forward-looking. However, enterprises with high quality can effectively transfer and allocate resources to innovative projects and obtain more considerable long-term benefits by increasing R&D investment [36]. In addition, this paper also proposes a possible explanation. Looking at historical development, the release of economic policies is determined by the government according to the economic environment at that time. The uncertainty of economic policies in periods of stable economic operation is generally low; During economic downturns and recessions, the government needs to change policies frequently and make more attempts to get out of financial difficulties [16], so economic policy uncertainty increases. From the perspective of enterprises facing relatively unfavorable external business conditions such as weak market demand, they naturally expect that the government will actively act, improve the investment environment, support the development of enterprises, and believe that the relevant economic policies in the future are good. And therefore, it will increase R&D investment [3].

To explore how economic policy uncertainty affects corporate R&D decisions, this paper takes three influence channels as the starting point for analysis, and Table 3 shows the specific empirical results. Models (1), (2), and (3) are the independent effects of cash flow uncertainty, product market competition, and debt financing channels, while model (4) adds three-channel variables simultaneously for regression analysis. It can be seen from model (4) that economic policy uncertainty is significantly positively correlated with corporate R&D investment (the correlation coefficient is 0.0019). From a financial point of view, monetary policy uncertainty (EPU) increases by one standard deviation; the average R&D investment of enterprises increased by 0.48%, which is a 10.8% increase in the sample average.

**Table 3:** Impact of economic policy uncertainty on firms’ innovation investments through three channels.

Dependent variable: *R*&*D*	(1)	(2)	(3)	(4)	(5)
	CFV Channel	HHI Channel	Debt Channel	Three channels	
*EPU*	0.0015***	0.0024***	0.0019***	0.0019***	0.0022***
	-3.276	-4.375	-3.984	-3.776	-4.414
*CF*	-0.0338			-0.0336	-0.0303
	(-1.57)			(-1.57)	(-1.51)
EPU*CFV	0.0160***			0.0162**	0.0158**
	-3.116			-3.246	-3.144
*HHI*		0.0394***		0.0397***	0.0397***
		-4.385		-4.446	-4.384
EPU*HHI		-0.0059**		-0.0062**	-0.0061**
		(-2.06)		(-2.15)	(-2.02)
*Debt*			-0.0213**	-0.0225**	
			(-2.69)	(-2.80)	
EPU*Debt			0.0082	0.0067	
			-0.254	-1.536	
*CF*	0.0085**	0.0096***	0.0088***	0.0070***	0.0170**
	-3.446	-3.585	-3.444	-3.246	-2.664
EPU*CF					-0.0030**
					(-2.10)
*Size*	0.0155***	0.0154***	0.0155***	0.0156***	0.0156**
	-3.886	-3.885	-3.844	-3.866	-3.884
*Age*	-0.0082**	-0.0082***	9.0084**	-0.0084***	-0.0083***
	-4.226	(-4.28)	(-4.21)	(-4.22)	(-4.27)
*Sale*	-0.0157**	-0.0156**	-0.0157**	-0.0159**	-0.0158***
	(-4.81)	(-4.81)	(-4.72)	(-4.76)	(-4.82)
*Lev*	-0.0290**	-0.0295***	-0.0280**	-0.0273***	-0.0290***
	(-4.86)	(-4.98)	(-5.35)	(-5.34)	(-4.89)
*Tang*	0.0125	0.0143	0.0161	0.015	0.007
	(-1.16)	(-1.36)	(-1.39)	(-1.57)	(-1.43)
*TobinQ*	0.0036***	0.0037***	0.0037***	0.0036***	0.0036**
	-3.156	-3.185	-3.194	-3.256	-3.244
*ROA*	0.017	0.0106	0.0149	0.0152*	0.0181
	-1.536	-0.735	-1.134	-1.756	-1.494
*DGDP*	-0.1527**	-0.1866***	-0.1483**	-0.1929**	-0.1892***
	(-2.62)	(-3.78)	(-2.47)	(-4.03)	(-3.88)
Constants	0.0573**	0.0545**	0.0588**	0.0589**	0.0556**
	-2.476	-2.455	-2.654	-2.766	-2.504
Industry effect	Yes	Yes	Yes	Yes	Yes
Year effect	Yes	Yes	Yes	Yes	Yes
Sample size	13435	13435	13435	13435	13435
R^2^	0.441	0.443	0.443	0.442	0.446

Note: Values in parentheses are t-values

***, **, * indicate significant at 1%, 5%, 10% confidence level, respectively.

From the channel of cash flow uncertainty, the coefficient of the interaction term (EPU*CFV) between economic policy uncertainty and cash flow uncertainty is significantly positive, indicating that cash flow uncertainty strengthens the positive impact of EPU on R&D investment, that is to say, EPU can improve the future profitability of innovation projects by increasing the cash flow uncertainty faced by enterprises, thereby promoting enterprises’ innovation investment and playing a "funding effect." This result validates H1. This result has also been confirmed in some literature. Combining the above literature review and theoretical analysis, this paper believes that the cash flow uncertainty not only means the risk of investment projects but also implies the opportunity to seize the market. This is because companies with forward-looking and economic intuition and operating solid capabilities are more likely to take potential opportunities through surface risks and have the ability and willingness to bear the possible negative impact of R&D investment. Therefore, the innovation investment of enterprises has an incentive effect [15]. Furthermore, listed companies generally have substantial capital and strength and are forward-looking companies with a high market share. Therefore, the "winner effect" is usually significant. Thus, the impact of cash flow uncertainty on innovation investment is mainly reflected in the "funding effect" [37].

From the perspective of product market competition channels, the coefficient of economic policy uncertainty and the interaction term of product market competition (EPU*CFV) is significantly negative, indicating that the higher the concentration of the industry in which the company is located, the more intense the competition, and the greater the uncertainty of economic policy. On the other hand, the greater the positive effect on enterprise R&D investment, that is, monetary policy uncertainty increases enterprise innovation investment by intensifying product market competition. This result validates H2. The reason for this result, combined with the literature review and theoretical analysis above, this paper believes that product market competition can stimulate the internal innovation power of enterprises. Some literature believes that in a competitive environment with serious homogeneity and numerous opponents, enterprises must increase their innovation efforts if they do not want to be buried by the tide of the times.

On the one hand, they can reduce costs through intelligent technology, and on the other hand, through new Products, break the phenomenon of market homogeneity, build competitive advantages with differentiated products, expand business channels and market demands, and turn passive defense into active competition [4]. Secondly, product market competition can improve enterprises’ innovation capabilities. Finally, due to the solid positive externalities of manufacturing product technology, market competition will bring knowledge spillover effects, a positive phenomenon for relatively backward enterprises. Enterprises can use this to acquire technical knowledge [23].

From the perspective of debt financing channels, debt financing is significantly negatively correlated with corporate R&D investment. This may be because more debt will reduce the innovation motivation of corporate managers, thereby reducing corporate R&D expenditures. This is consistent with the control variable asset-liability ratio in the model—the coefficient before (Lev) (-0.026 is significantly harmful. The coefficient of the interaction term between economic policy uncertainty and debt financing (EPU*Debt) is not significant, indicating that monetary policy uncertainty will not pass Affecting debt financing has an impact on corporate R&D investment; that is, debt financing is not a channel through which economic policy uncertainty affects corporate R&D investment. The possible explanation is that debt financing decisions are relatively short-term, adjustable, and more. It is used to deal with the enterprise’s daily operation or general investment, and enterprise innovation is a reasonably long-term decision. At the early stage of decision-making, managers will consider the possible borrowing difficulties in the future because the enterprise’s innovation investment projects have high risks and low monitoring. Information asymmetry is severe; it is difficult to obtain credit support from banks, so they will be more inclined to internal financing. To test this idea, this paper uses the cash flow of business activities to measure the level of internal financing, adding economic policy uncertainty and operationality. The interaction term of cash flow (EPU*CF) is tested by model (5) regression in Table 3. From the regression results of model (5), we can see that internal cash flow has a positive effect on the R&D investment of enterprises, which indicates that enterprises’ R&D investment depends on internal financing. However, the interaction coefficient is significantly negative, meaning that higher economic policy uncertainty will weaken the positive effect of internal financing on enterprise R&D investment. Financing is blocked so that part of the internal funds is converted into daily operations or general investment, thus reducing R&D investment [13]. Therefore, economic policy uncertainty reduces the positive effect of internal financing on the R&D investment of enterprises. Instead of using the external debt financing channel, we initially assumed.

### 5.2. Impact of economic policy uncertainty on the effect of R&D investment

This paper also explores the effect of economic policy uncertainty on R&D investment, i.e., innovation output. The focus of previous research in this paper has been on the input side of innovation investment. Still, the effect of investment, i.e., R&D output, is also worthy of attention and can reflect the effectiveness of R&D investment to a certain extent. Therefore, this paper uses the number of patent applications to measure the R&D output of enterprises, including the number of invention patents, utility model patents, and design patents. The number of patent applications (Patent) is used as the explanatory variable. Economic policy uncertainty (EPU) with one lag and R&D investment (R&D) with one lag are used as explanatory variables in the regression analysis to investigate the impact of economic policy uncertainty on R&D output. The results are shown in Table 4.

**Table 4:** Impact of economic policy uncertainty on R&D output.

Dependent variable: *R*&*D*	Full sample	2012–2015	2016–2020
	(1)	(2)	(3)
*EPU*	0.027	0.048*	-0.022*
	(1.56)	(6.49)	(-4.62)
*R*&*D*	0.146***	0.126**	0.142**
	(2.58)	(2.86)	(2.38)
EPU*R&*D*	-0.086*	-0.164*	-0.038**
	(-1.68)	(-1.48)	(2.26)
Industry Effect	Yes	Yes	Yes
Year Effect	Yes	Yes	Yes
Sample Size	13435	7057	6378
R^2^	0.143	0.140	0.137

Note: Values in parentheses are t-values

***, **, * indicate significant at 1%, 5%, 10% confidence level, respectively.

From model (1) in Table 4, it can be seen that the R&D input in the previous period has a significant contribution to the R&D output in the current period. To a certain extent, it also reflects the conversion rate of innovation input and output, but in general, the effect of economic policy uncertainty on R&D output is not significant. Models (2) and (3) in Table 4 regress the sample into two subsamples, 2012–2015 and 2016–2020, based on the magnitude of the EPU index values, and find that economic policy uncertainty has a positive contribution to firms’ R&D output when monetary policy uncertainty is low (i.e., 2012–2015), and when economic policy uncertainty is high (i.e., 2016–2019), which is consistent with the findings of [38]. In addition, the coefficient of the interaction term (EPU*R&D) between economic policy uncertainty and R&D input is negative in all three models, indicating that the higher the tension, the less the positive contribution of R&D input to patent output. Hence, although economic policy uncertainty promotes the amount of R&D input of firms, it weakens the utility of R&D input to encourage output patent, reduces the conversion rate of innovation inputs and outputs, and decreases the R&D activities, i.e., the effect of R&D inputs may not be good.

### 5.3. Endogenous issues

Regarding the discussion of endogeneity, since the state formulates economic policies, it is almost impossible for individual firms to influence economic policies, and all explanatory variables in the empirical process use a one-period lag, there is no reciprocal causality between the dependent variables and the leading independent variables in this paper. Therefore, this paper argues that the possible endogeneity in the study arises from omitted variable errors, i.e., there is a correlation between the significant explanatory variables and the omitted variables. This paper strictly controls for the year and industry effects in the empirical study and also regresses subsamples that are similar at the firm or industry level (e.g., whether they are high-tech firms, whether they have a political affiliation, etc.) in the heterogeneity analysis, which avoids the omitted variable problem to a certain extent and mitigates the resulting endogeneity problem.

In addition, this paper also refers to [39]. It uses systematic generalized moments estimation (GMM) to alleviate the following two problems:(1) generalized moments estimation is mainly applicable to dynamic panel estimation, considering that innovation input behavior is a long-term decision behavior. There is a large stickiness of R&D input in two adjacent periods, i.e., R&D input in the current period is likely to depend mainly on the previous period R&D. The current period’s R&D input is expected to rely primarily on the last period’s R&D input level. Therefore, this paper adopts dynamic panel regression, which can increase the robustness of the regression results. (2) Generalized moment estimation can mitigate the possible endogeneity problem through instrumental variables. This paper selects the global EPU index with two lags and the Chinese EPU index with two lags as instrumental variables for the Chinese EPU index with one lag.

Table 5 reports the GMM regression results, consistent with the benchmark regression results in Table 3. Still, it can be seen that the previous period’s R&D investment has a significant impact on the current period’s R&D investment, which is consistent with the investment stickiness of innovation investment and also illustrates side-by-side that a firm’s R&D investment is a long-term behavior that cannot be easily decoupled from the firm’s development strategy. Although the empirical results in Table 5 show that the absolute values of the coefficients before several main explanatory variables have decreased. The significance of the coefficients before the three-channel interaction terms has also been reduced. Nevertheless, the overall results are consistent with the results of the benchmark regression above. The effect of EPU on R&D investment is still significant at both the statistical and economic significance levels.

**Table 5:** Static panel and dynamic panel regression results.

Dependent variable: R&D	Static model	Dynamic Model
*EPU*	0.0068***	0.0058***
	(4.63)	(3.76)
EPU*CFV	0.0186***	0.0118**
	(3.68)	(1.76)
EPU*HHI	-0.0151**	-0.0048***
	(-2.62)	(-4.75)
EPU*CF	-0.0068**	-0.0075*
	(-2.38)	(-1.48)
*CFV*	-0.0336	0.0182
	(-1.46)	(1.68)
*HHI*	0.0375***	0.0162***
	(4.63)	(3.78)
*CF*	0.0136**	0.0184**
	(2.63)	(2.84)
*R*&*D*_*t*−*I*_		0.734***
		(36.45)
*R* ^2^	0.440	
*AR*(1)*test*(*p*−*value*)		0.000
*AR*(2) *test*(*p*-*value*)		0.376
*Hansen test of over-identification* (*p*-*value*)		0.140
*Diff-in-Hansen tests of exogeneity* (*p*-*value*)		0.556

Note: Values in parentheses are t-values

***, **, * indicate significant at 1%, 5%, 10% confidence level, respectively.

### 5.4. Other robustness tests

(1) Changing the explanatory variables

In the robustness test, this paper changes the calculation of the explanatory variables to R&D investment/total assets at the beginning of the period and R&D investment/((total assets at the beginning of the period + total assets at the end of the period)/2), repeats the benchmark regression above. The results are consistent with the above (the regression results are not given in this paper due to space limitation).

(2) Changing the EPU index

Since the frequency of the EPU index constructed by Baker et al. is measured every month, and the other corporate financial data in this paper are annual, it is necessary to transform the monthly data into a yearly index. The relevant empirical tests above all use annual EPU indices calculated by weighted averaging. This paper also tries different transformations (including arithmetic and geometric averaging) to construct annual economic policy uncertainty. The results of the benchmark regressions are generally consistent with Table 3 (the regression results are not given in this paper due to space constraints).

In addition to [13, 40] also constructs an EPU index based on a similar approach, and Table 6 shows the differences between the three EPU indices. In this paper, the economic policy uncertainty indices constructed by [37, 41] are used in the robustness tests, respectively, and the baseline regressions above are repeated, and the results are also generally consistent with Table 3 (regression results are not given in this paper due to space constraints).

**Table 6:** Comparison of the three EPU indices.

	[42]	[43]	[38]
News search based on the newspaper	South China Morning Post, Hong Kong	People’s Daily and Guangming Daily	Beijing Youth Daily, Guangzhou Daily, Jiefang Daily, People’s Daily Overseas, Shanghai Morning Post, Southern Metropolis Daily, Xinjing Daily, Today’s Evening Post, Wenhui Daily, Yangcheng Evening News

## 6. Conclusions and recommendations

### 6.1. Conclusion

This paper describes the theoretical mechanism of economic policy uncertainty affecting corporate R&D investment. It explores the impact of monetary policy uncertainty on corporate R&D investment and its channels through empirical analysis using A-share corporate R&D data and the China EPU index. The theoretical hypothesis section of this paper argues that the degree and direction of the impact of economic policy uncertainty on firms’ innovation investment differ through different channels, and there is an interaction effect. Therefore, based on the investment decision principle of fundamental options theory, we investigate the impact of EPU on innovation investment through three channels: cash flow uncertainty, product market competition, and debt financing from the three main factors that affect investment—investment return, waiting for value, and investment cost, and the findings are as follows.

First, economic policy uncertainty has a positive incentive effect on firms’ R&D investment. In general, the increase in economic policy uncertainty is a positive signal for firms’ R&D, effectively promoting them to invest in R & R&D. This positive relationship seems to be somewhat unexpected. Still, through the analysis in the hypothesis section of the study and the context of China’s current actual economic situation, this optimistic, positive effect is justified: in the past, China had a large gap with the world’s frontier technology, and firms could enter the market at a lower cost by simply imitating the technology through the knowledge spillover effect, which caused severe homogenization of firms and intensified product market competition. And in the present time, accelerating product and technology innovation has become the only rule for enterprises not to be eliminated or even stand out in the hundred boats competing in the industry. In such a competitive context, economic policy uncertainty poses challenges and implies opportunities for future increased returns. Thus, despite its adverse effects on firms’ general and physical capital investments, rational and forward-looking firms can effectively shift and allocate resources to innovation projects to obtain more substantial long-term returns by increasing R&D investments. In addition, this paper also proposes a possible explanation that the government side issues economic policies according to the prevailing economic environment and that monetary policy uncertainty increases during economic downturns and recessions when policymakers need to change economic policies frequently to get out of financial difficulties. On the other hand, Enterprises naturally hold the optimistic expectation that the government will act positively, improve the investment environment and support the development of enterprises at this time, and consider the relevant economic policies to be favorable in the future so that they will increase their R&D investment.

Second, this paper explores the impact of economic policy uncertainty on enterprise R&D through three channels. The main findings are summarized as follows: (1) Economic policy uncertainty promotes R&D investment through the cash flow uncertainty channel, and when enterprises face cash flow uncertainty brought by high economic policy uncertainty, they attach more importance to the opportunities and future profitability, which motivates them to increase their R&D investment. (2) Economic policy uncertainty promotes R&D investment through the product market competition channel. When economic policy uncertainty is high, each enterprise subject considers it an excellent time to seize the opportunity, and postponing R&D investment will instead lose the chance to take the market, especially in industries with high product market competition; the more significant this positive promotion effect is. (3) The debt financing channel does not play a role. A possible explanation is that although economic policy uncertainty raises the cost of debt financing, firms’ innovation investment mainly relies on internal financing, so economic policy uncertainty does not directly affect R&D investment through the debt financing channel. Based on this, this paper makes a related empirical verification and finds that firms’ external financing is blocked when economic policy uncertainty is high. Firms thus convert part of their internal funds to the daily operation or ordinary investment, so economic policy uncertainty reduces firms’ innovation investment by weakening the positive effect of internal financing on firms’ R&D investment, rather than directly through external debt financing channel.

### 6.2. Recommendation

The research content and empirical results of this paper have strong policy implications for the real world today, mainly in two ways.

(1) Policy introduction or change should fully consider the possible effects of economic policy uncertainty on each economic agent. Although studies have concluded that economic policy uncertainty has a positive impact on R&D investment, some subsequent studies have also shown the possible adverse effects of economic policy uncertainty. Therefore, when relevant departments introduce or adjust economic policies, they should stabilize the market economic environment, effectively support enterprises’ investment and financing activities, help them to tide over difficulties, respond positively to meet their positive expectations, improve high-tech enterprises’ confidence in macroeconomic policies, and provide a favorable external policy environment for their technological R&D and product innovation.

(2) Relevant departments should be committed to building a favorable external environment to mobilize enterprises’ R&D enthusiasm and make them burst into innovation. The research in this paper shows that the greater the incentive impact of economic policy uncertainty in industries with high degree of product market competition. Therefore, while enterprises themselves actively adapt to product market competition, the government should establish a reasonable competition mechanism and shape a good competition environment so that product market competition can play its external governance role and properly guide and encourage enterprises to participate in market competition. This paper also concludes that when economic policy uncertainty is high, external financing is hindered and a portion of internal funds is converted to daily operations or general investments, thus curtailing R&D investment. Therefore, the government should be committed to developing and improving financial markets to broaden external financing channels, especially for R&D projects, which can be done through joint loans from the government, banks, and insurance, so that enterprises’ R&D no longer relies mainly on internal financing, thus releasing innovative energy.

### 6.3. Research deficiencies and prospects

(1) Restrictions on R&D data. At present, the relevant accounting policies for companies to disclose R&D expenses are not perfect. On the one hand, there is no mandatory requirement for this disclosure, and on the other hand, there is no uniform standard for data disclosure. Therefore, the research and development data collected in this paper through the database may be defective, affecting the research results.

(2) The limitations of sample selection. Since there are many missing values in the data of non-listed companies, the continuity of corporate data is not strong, and there is no uniform standard for R&D data, there are many outliers, so this paper selects the data of listed companies. However, listed companies generally have strong operating capabilities, have a strong cash flow to support R&D investment, and have greater strength to bear the risk of failure of R&D investment. Therefore, using listed companies as a sample for regression, the conclusions drawn may only apply to large enterprises with good operating conditions, not to unlisted enterprises, nor to represent the general behavior of Chinese enterprises. Of insufficiency.

(3) The validity of the EPU index. On the one hand, the EPU index is artificially constructed, and the value lacks a specific economic meaning. It can only reflect the relative degree of monetary policy uncertainty through its size. Therefore, the research conclusion lacks quantitative guidance. On the other hand, although the existing literature has confirmed that the EPU index can effectively measure economic policy uncertainty from a theoretical and empirical point of view and is widely used in related research, the index may still have measurement errors. It may also contain some Policy-irrelevant macroeconomic uncertainties, thereby affecting research conclusions.

Given the above shortcomings, this paper puts forward the prospect of future research: first, improve the relevant rules and regulations of R&D expense disclosure to ensure the accuracy of enterprise R&D data; second, the data of the Chinese industrial enterprise database can be used as a sample for future research. Research can also compare the heterogeneous behaviors of listed and non-listed companies in response to economic policy uncertainty to draw broader and comprehensive conclusions that can represent general corporate behavior; third, in future research, some measures can be used. The method removes the macroeconomic uncertainties contained in the EPU index or compares the impact of the two delays on R&D investment to ensure the robustness of the empirical results.

## Supporting information

S1 Dataset (XLSX)
